# Ocean acidification as a driver of community simplification via the collapse of higher-order and rise of lower-order consumers

**DOI:** 10.1038/s41598-017-03802-w

**Published:** 2017-06-22

**Authors:** S. Vizzini, B. Martínez-Crego, C. Andolina, A. Massa-Gallucci, S. D. Connell, M. C. Gambi

**Affiliations:** 1 Department of Earth and Marine Sciences, University of Palermo, Palermo, Italy; 2 CoNISMa, Roma, Italy; 3 Centre of Marine Sciences (CCMAR), Faro, Portugal; 4 Department of Environmental Sciences, Informatics and Statistics, DAIS, University Ca’ Foscari, Venice, Italy; 5 Stazione Zoologica Anton Dohrn, Department of Integrative Marine Ecology, Villa Dohrn Benthic Ecology Center (Ischia), Naples, Italy; 6 Southern Seas Ecology Laboratories, School of Biological Sciences & Environment Institute, University of Adelaide, South Australia, Australia

## Abstract

Increasing oceanic uptake of CO_2_ is predicted to drive ecological change as both a resource (i.e. CO_2_ enrichment on primary producers) and stressor (i.e. lower pH on consumers). We use the natural ecological complexity of a CO_2_ vent (i.e. a seagrass system) to assess the potential validity of conceptual models developed from laboratory and mesocosm research. Our observations suggest that the stressor-effect of CO_2_ enrichment combined with its resource-effect drives simplified food web structure of lower trophic diversity and shorter length. The transfer of CO_2_ enrichment from plants to herbivores through consumption (apparent resource-effect) was not compensated by predation, because carnivores failed to contain herbivore outbreaks. Instead, these higher-order consumers collapsed (apparent stressor-effect on carnivores) suggesting limited trophic propagation to predator populations. The dominance of primary producers and their lower-order consumers along with the loss of carnivores reflects the duality of intensifying ocean acidification acting both as resource-effect (i.e. bottom-up control) and stressor-effect (i.e. top-down control) to simplify community and trophic structure and function. This shifting balance between the propagation of resource enrichment and its consumption across trophic levels provides new insights into how the trophic dynamics might stabilize against or propagate future environmental change.

## Introduction

As a consequence of increasing CO_2_ emissions in the atmosphere, oceanic uptake of CO_2_ is predicted to rise progressively^1, 2^ with concomitant changes to pH and carbonate chemistry affecting marine organisms^3, 4^ and their ecosystem functions^4^. In recent recognition of the potential extent of this change, there has been a sudden increase in ecological research concerning ocean acidification (OA). Initially, research focused on CO_2_ enrichment in laboratory and mesocosm experiments (e.g. ref. 5), progressing from physiological and morphological responses of individual species through community^6, 7^ and ecosystem^8^ level responses. Conceptual models anticipate that near future concentrations of CO_2_ may be severe for calcifying organisms (i.e. OA acts as a stressor), while boosting growth and photosynthesis in fleshy algae and seagrasses (i.e. CO_2_ acts as a resource)^4, 6, 9^. As a result, major ecosystem disruption has been almost universally inferred at high CO_2_^3^, although this assumption remains largely untested^10^.

The assessment of processes that propagate or buffer change is challenging in simplified laboratory and mesocosm research. Recent focus has incorporated natural systems where volcanic CO_2_ emissions naturally acidify coastal waters^9, 11–15^. A relatively large number of studies at the Castello Aragonese CO_2_ vent of Ischia Island (Italy, Tyrrhenian Sea) have begun to shed light on the long-term biological and ecological responses along pH gradients at varying levels of biological hierarchy, from species-specific responses^16–18^ to patterns of motile invertebrates^19, 20^ and macroalgae assemblages^21^. Of increasing interest from the global study of CO_2_ vents has been the reported increase in the abundance of non-calcifying macroalgae that boost herbivore biomass^9, 22, 23^, suggesting that trophic compensation might buffer boosted primary productivity^24^ (i.e. stabilizing mechanisms^25^). These studies suggest that complex communities are worth more attention, particularly because less attention has been paid to OA effects on trophic and functional diversity and ecosystem functioning (but see refs 4, 8, 20 and 26).

Trophic complexity of communities has been widely investigated using stable isotope analysis, however this powerful tool has been rarely used to investigate the food web structure and trophic diversity in volcanic CO_2_ vents (but see ref. 27). Such analyses may provide valuable insights into the predicted effects of OA on naturally complex food web dynamics, which suggest trophic plasticity in herbivores that maintain food intake despite changes in nutritional quality of primary producers^27^. Such trophic flexibility to OA-altered primary productivity indicates that complex communities have compensatory dynamics that enable them to withstand the intensifying effects of abiotic change^8, 10^.

Ecological theory recognises that abiotic change can drive declines in biodiversity and trophic diversity^28^ and a subsequent loss of ecosystem productivity and stability^29^. Yet, at moderate levels of abiotic change, compensatory effects may absorb disturbance and promote stability^25, 30^ (e.g. trophic compensation^24^). Abiotic drivers of bottom-up and top-down change in community dynamics are reflected in changes in food web structure, with the intensity of such changes often depending on how population densities vary with biodiversity^30–32^. Persistent abiotic change that boosts the productivity of primary producers (i.e. bottom-up forcing) might not include only shorter food webs, but also simpler food webs that favour opportunistic species (e.g. herbivores and detritivores^33^). Such simplification is predicted to reduce energy flow to higher trophic levels (i.e. predators) and their top-down control^33, 34^, such that herbivore populations boom^9, 22^. Hence, CO_2_ can also act as a resource to primary producers^23^ and their consumers^9, 22^, which moderates the effects of OA^10^. Such trophic changes and feedbacks are ubiquitous among systems in which human activity alters top-down and bottom-up processes^29, 31, 34, 35^, but there remains little insight into how they may change as a function of increasing human CO_2_ emissions^8^.

We test predictions of change by observing the community and food web structure at a CO_2_ vent at Vulcano Island (Italy, Tyrrhenian Sea). Whilst spatial replication at this site is limited to the comparison of contemporary and enriched *p*CO_2_ levels (i.e. control *vs*. vent sites), it does offer an opportunity to assess the potential validity of existing concepts for which the discipline has limited ability to assess with laboratory and mesocosm approaches. In particular, we tested the hypotheses that CO_2_ enrichment (1) leads to greater biomass of primary producers of higher nutritional quality, which amplify their trophic role for consumers (i.e. CO_2_ as a resource-effect), but (2) reduces biodiversity of invertebrates because the costs of the OA (i.e. stressor-effect) outweigh the benefits of food with higher nutritional values, such that (3) the combined effect of enriched resources (boosting bottom-up processes) and intensifying stress (dampening top-down processes) results in shorter food web and reduced trophic diversity.

## Results

### Composition and food quality of primary producers

Macrophyte composition differed between the study sites (see Supplementary Fig. 2S), where the control included two seagrass species (*Cymodocea nodosa* and *Zostera noltei*), and two macroalgae species (*Cystoseira compressa* and *Padina pavonica*). At the CO_2_-enriched site, *Z*. *noltei* and *Padina pavonica* were absent, while *C*. *nodosa* dominated the macrophyte community, followed by the macroalgae *Dictyota dichotoma*, *Caulerpa prolifera* and *C*. *compressa*. In addition, unidentified filamentous algae were found to be abundant at both sites. Despite the large variability, overall macrophyte biomass was significantly smaller at the control site (t-test p = 0.003). Epiphyte biomass was not detected to differ between sites, but calcareous epiphytes only occurred at the control site.

**Figure 1:**
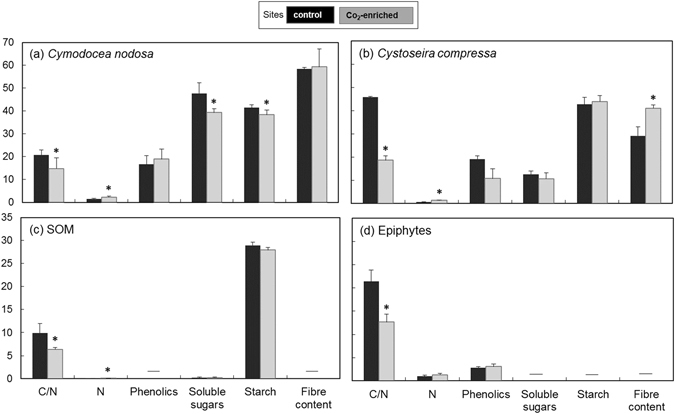
Chemical features indicative of the nutritional quality of food sources present in both control (black) and CO_2_-enriched (grey) sites. C/N ratio, N (%), phenolics (mg g^−1^), soluble sugars (mg g^−1^), starch (mg g^−1^) and fibre (%) content of (**a**) *Cymodocea nodosa*, (**b**) *Cystoseira compressa*, (**c**) sedimentary organic matter – SOM, and (**d**) epiphytes. All bars indicate standard deviation. — indicates data not available. Asterisks indicate t-test’s p-value < 0.05.

Comparing the nutritional quality of food sources found in both sites, C/N ratio decreased and N increased from the control to the CO_2_-enriched site in *C*. *nodosa*, *C*. *compressa* and the sedimentary organic matter, while only C/N ratio decreased in epiphytes (Fig. 1). No significant changes were observed in phenolic content, which showed a low concentration in epiphytes at both sites. Carbohydrates (soluble sugars and starch) were both significantly higher at the control site than at the CO_2_-enriched site for *C*. *nodosa*, while they showed no difference for *C*. *compressa*. Fibre content significantly decreased at the control site in *C*. *compressa*.

### Community structure and biodiversity of the motile invertebrate community

Overall, we sampled and identified a total of 1263 individuals of motile invertebrates belonging to 44 taxa, with 32 taxa (384 individuals) found at the control site, and 23 taxa (879 individuals) found at the CO_2_-enriched site (see Supplementary Table 1S). The two study sites had a total of 11 taxa in common, with Sorensen similarity index of 0.40.

The main taxa found were Crustacea Peracarida (amphipods, isopods and tanaids, 14 taxa), Mollusca (especially gastropods, 12 taxa) and Polychaeta (9 taxa) (Table 1S).

The nereidid *Platynereis dumerilii*, a species commonly associated with macrophytes (both algae and seagrasses)^36^, represented 90% of the Polychaeta class. We have classified this species as *P*. cfr *dumerilii* given that the taxonomic identity of the *P*. *dumerilii* population is currently being further investigated using genetic analysis due to the recent discovery of a sibling species of this taxon at the Vulcano vent^37, 38^. Most of the individuals (98%) were collected at the CO_2_-enriched site.

**Figure 2:**
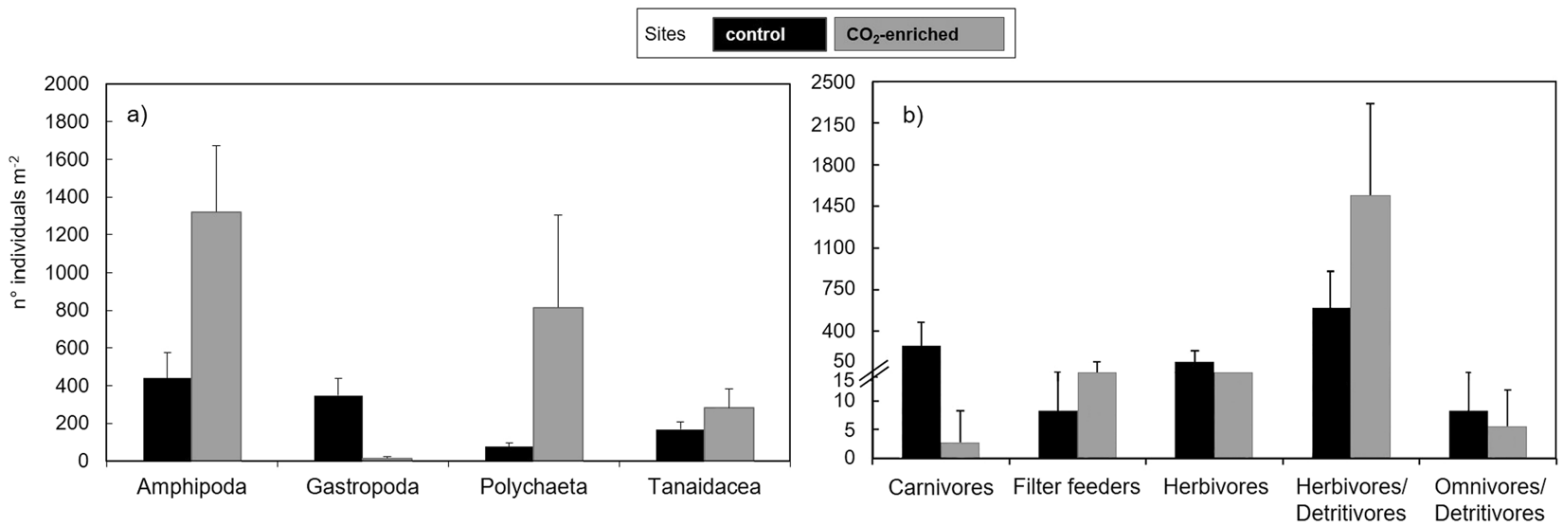
Abundance of the motile invertebrate taxa and trophic groups at the control (black) and CO_2_-enriched (grey) sites. Density (n° individuals m^−2^; mean ± standard error) of (**a**) main taxa and (**b**) trophic group of the motile invertebrate community.

Mollusca were numerically dominated by gastropods which were almost entirely collected in the control site (Table 1S, Fig. 2a) and consisted of 74% *Bittium reticulatum*.

Crustacea Peracarida included mainly Amphipoda, represented by various species belonging mainly to Caprellidae (*Phtisica marina* and *Caprella acanthifera*) and other families (e.g. Ischyroceridae), and Tanaidacea, almost entirely represented by *Chondrochelia savignyi* (Table 1S).

At the CO_2_-enriched site, many taxa were observed at greater density (11-fold for Polychaeta, 3-fold for Amphipoda and 2-fold for Tanaidacea), whilst Gastropoda were sparser (Fig. 2a) and showed shell corrosion.

Shannon and Pielou indexes revealed greater diversity at the control site, with a mean increase from 1.36 to 1.96 in H’ (t-test p = 0.003) and from 0.61 to 0.77 in J’ (t-test p = 0.051) compared to the CO_2_-enriched site. When considered as trophic groups, herbivores/detritivores numerically dominated the control site (Fig. 2b), so that they represented the majority of taxa (58.4%), followed by carnivores (24.7%) and herbivores (14.6%), while other groups (omnivores/detritivores and filter-feeders) were a much smaller proportion (1.5% and 0.8% respectively). Fewer trophic groups contributed to the community structure at the CO_2_-enriched site, which was dominated by herbivores/detritivores (72.9%) and herbivores (25.5%), while filter-feeders accounted for only 1.1% and omnivores/detritivores and carnivores were almost absent (0.4% and 0.1% respectively).

### Isotopic composition, food web structure and trophic diversity

**Figure 3:**
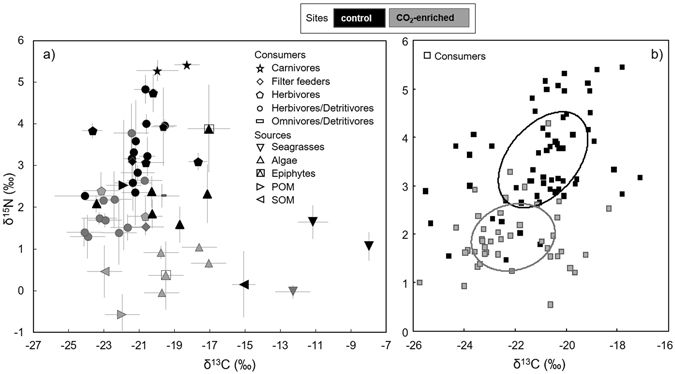
Isotopic signatures at the control (black) and CO_2_-enriched (grey) sites. (**a**) δ^13^C *vs*. δ^15^N (‰, mean ± standard deviation) of each organic matter source (POM: particulate organic matter, SOM: sedimentary organic matter) and consumers (assigned to trophic groups). (**b**) δ^13^C *vs*. δ^15^N (single replicates) of consumers: solid lines enclose the standard ellipse areas (SEAc).

Overall, basal food sources (i.e., seagrasses, algae, epiphytes, POM and SOM) presented both ^13^C and ^15^N-depleted signatures in the CO_2_-enriched site (Fig. 3a). δ^13^C ranged between −23.56‰ and −7.82‰ and between −24.13‰ and −11.02‰ in the control and CO_2_-enriched sites respectively, while values of δ^15^N were much narrower, from 0.15‰ to 3.89‰ at the control site and from −0.57‰ to 1.04‰ at the CO_2_-enriched site.

A comprehensive look at the species distribution of the motile invertebrates within the isotopic space revealed a clear shift in the isotopic niche towards lower δ^15^N, and to a lesser extent δ^13^C, from the control to the CO_2_-enriched site (Fig. 3a,b). In addition, niche width was narrower at the CO_2_-enriched site (Fig. 3b), as reflected by both δ^15^N range (NR) and δ^13^C range (CR) metrics, which ranged from 2.49‰ to 3.15‰ and from 4.41‰ to 6.43‰ respectively. Community-wide isotopic measures of trophic structure, quantifying trophic diversity and redundancy, such as Distance to Centroid (CD), mean Nearest Neighbour Distance (NND) and Standard Deviation of the Nearest Neighbour Distance (SDNND), did not show important variations between the study sites, varying from 1.42‰, 0.60‰ and 0.64‰ at the control site to 1.34‰, 0.50‰ and 0.35‰ at CO_2_-enriched site respectively. Corrected Standard Ellipse Area (SEAc), another measure of trophic diversity, changed in width, position and shape, decreasing from 4.84‰^2^ at the control site to 3.55‰^2^ at the CO_2_-enriched site and exhibiting almost no overlap between the two sites (0.05‰^2^).

**Figure 4:**
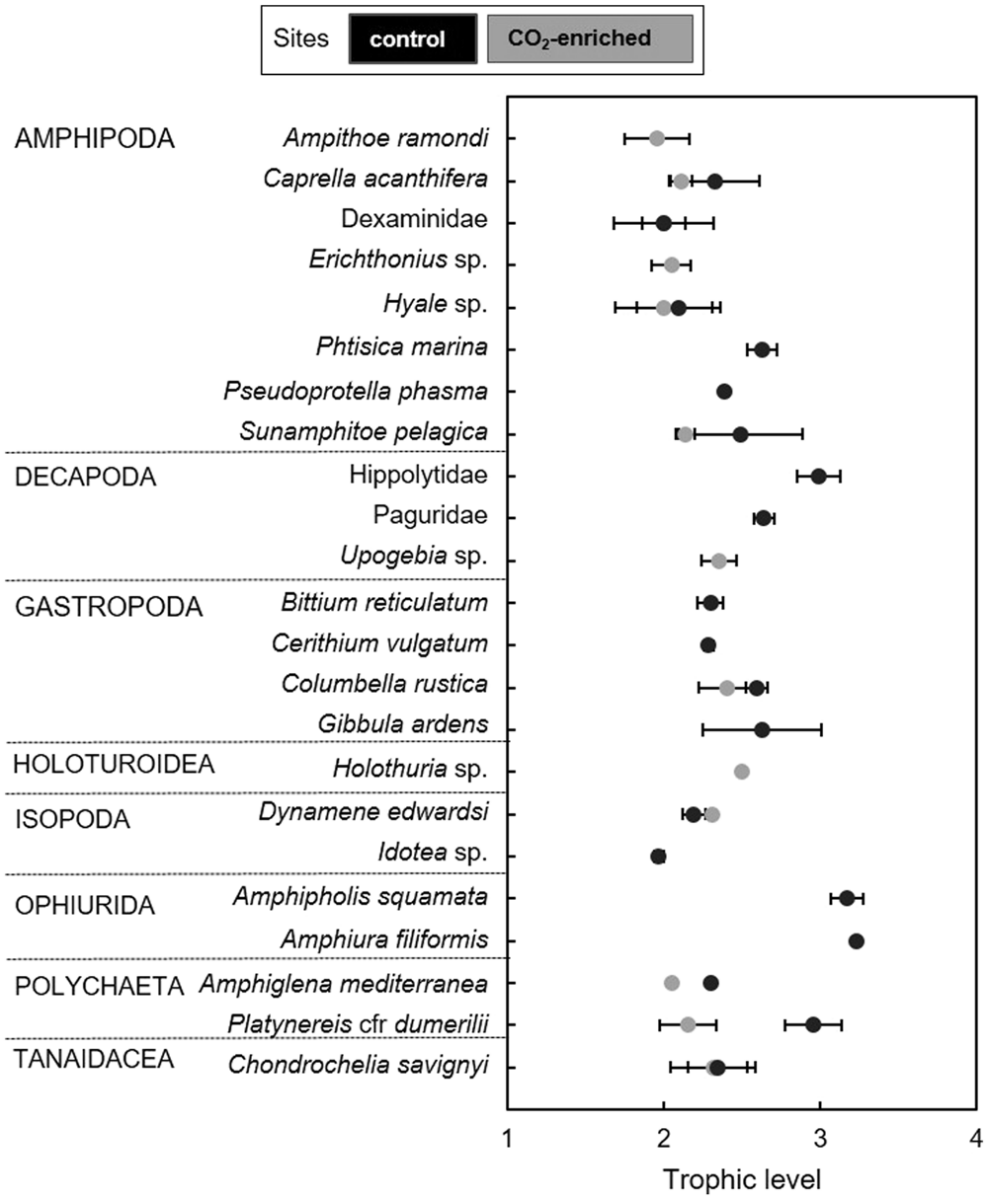
Trophic position (± standard deviation) of motile invertebrates associated with macrophytes at the control (black) and CO_2_-enriched (grey) sites.

All the species in common between the two sites occupied higher trophic levels at the control site (Fig. 4) than at the CO_2_-enriched site. The mean trophic level was slightly higher at the control site (2.50 ± 0.37 at the control; 2.18 ± 0.17 at the CO_2_-enriched site) and estimated trophic levels varied between 1.97 and 3.23 at the control site and between 1.96 and 2.50 at the CO_2_-enriched site.

Bayesian mixing model results for species found in both sites show only slight differences in source partitioning between the two environmental conditions, with some noteworthy aspects reported below. At the control site the contribution of the four food sources was homogeneous for all the consumer species (around 25%), and for *C*. *savignyi* and Hippolytidae only algae seemed to contribute more (35%) at the expense of SOM for *C*. *savignyi* and of both SOM and seagrasses for Hippolytidae. The proportions remained almost unvaried at the CO_2_-enriched site, although the strictly herbivorous *P*. cfr. *dumerilii*, *C*. *rustica* and *C*. *savignyi* showed an increase in consumption of SOM (respectively 44, 39, and 40%) at the expense of seagrasses for *P*. cfr. *dumerilii* and *C*. *rustica* and of algae for *C*. *savignyi*.

However, the low 95th percentile for most of the solutions was 0 or close to 0 (see Supplementary Table 2S), indicating a high variability in the probability of the likely contributions.

## Discussion

We tested the hypothesis that CO_2_ enrichment interacts with complex ecological communities as both a resource-effect and a stressor-effect (i.e. boosted primary productivity and herbivore responses). CO_2_ enrichment was associated with greater biomass of primary producers of higher nutritional quality, but lower biodiversity of motile invertebrates and numerical dominance of beneficiary species; i.e. boosted populations of herbivores and detritivores rather than carnivores. The resulting food web was shorter and trophically simpler. The shifting balance between the propagation of resources (affected by CO_2_ enrichment) and its consumption (affected by acidification and resource availability) reflects a chain of direct and indirect interactions within food webs.

First, the biomass of primary producers was greater at the site of elevated CO_2_ and associated with enhanced nutritional quality. Consequently, the abundance of herbivores and detritivores was boosted in association with these CO_2_-enriched basal sources, suggesting that the resource-effect of enrichment on primary producers carries through to their consumers. Herbivores are the indirect beneficiaries of CO_2_ enrichment *via* their feeding: the plants, their epiphytes or their detritus. Whilst the resource-effect is well documented *via* the direct effects on primary producers^23^ the extent to which greater rates of per capita consumption^39^ and populations of herbivores^22^ can compensate for greater primary production is largely unknown.

Our study provides some insight into the potential for herbivores to hold boosted production in check (i.e. trophic compensation^24^), suggesting that elevated production at near future CO_2_ levels may not be strongly buffered. Whilst it is common to observe greater productivity of filamentous algae within vents and mesocosms^23^ along with their herbivores and detritivores^9, 22^, as we also observed, the potential for trophic compensation appeared small. These observations suggest that boosted primary productivity at near future CO_2_ concentrations may well exceed the counterbalancing effect of herbivory^40^ in coastal marine ecosystems.

The propagation of CO_2_ enrichment to secondary productivity was profound. Assemblages living under enrichment were characterised by fewer taxa, primarily as a consequence of a marked reduction in Gastropoda, as predicted for the stressor-effect of OA on calcifying species^12^. While high and dense seagrass canopy (i.e. *Posidonia oceanica*) may act as a buffer on pH reduction and consequent OA effects^20^, this was not the case in our study, probably due to the small size and biomass of the seagrass species (*C*. *nodosa*), suggesting that the pH buffering effect of seagrasses is species-specific. Yet, as predicted by models of species replacement^6^, there was a switch to large numbers of no- or less calcified taxa (i.e. polychaete *P*. cfr. *dumerilii*, a few Caprellidae and other amphipods). This switch resulted in lower biodiversity, in line with the pattern observed in the invertebrate community associated with rocky reef algae in the CO_2_ vent located at Ischia Island^19^. If species are replaced by those with similar functional roles, then perhaps they can compensate for the loss of biodiversity^41^, but this remains a priority area for future research^30^.

Reduced abundance of motile carnivorous invertebrates was associated with elevated CO_2_. Higher-level consumers (i.e. secondary consumers) were nearly absent at the CO_2_-enriched site, at near-future CO_2_ concentrations. This scarcity of carnivores at elevated CO_2_ is consistent with previous observations (e.g. polychaetes^42^) in which elevated CO_2_ was related to greater abundance of herbivores and detritivores. Functional analysis of motile macroinvertebrates in seagrasses reveals a reduction in epiphyte biomass and leaf canopy height under elevated CO_2_, due to intense fish grazing^43^. Such switches in relative abundance of functional groups (i.e. increase of herbivore-detritivores and reduction in carnivores), with food web simplification, appears to be an emergent community property^44^ of high CO_2_ world^4^.

Boosted primary producer biomass (mainly macroalgae) and concomitant increase in their nutritional quality is quite general^45, 46^ (higher abundance of non-calcifying algae with higher N content and lower C/N ratios). This increase in availability of energy and nutrient appears to propagate to herbivores as a function of their increased feeding intensity on CO_2_-enriched plants^39^ to offset the costs of acidification^10^. Nevertheless, the extent to which this feedback can buffer future food webs from change is unknown. In our study, the entire food web shifted towards more ^13^C-depleted values: consumers mirrored the isotopic distortion of primary producers due to the exploitation of volcanic ^13^C-depleted nutrients and increased photosynthetic enzyme discrimination against ^13^C, also reported in previous studies under high CO_2_ conditions^13, 47^. However, any evident change in the trophic contribution of macrophytes to primary consumers was quantified, providing evidence that their trophic role was not amplified. Indeed, isotopic mixing models did not detect a more prominent role of primary producers in the diet of consumers at CO_2_-enriched sites, while the benthic food web was influenced approximately to the same degree by all the sources. Similarly, although focused on only three herbivorous polychaete species, Ricevuto *et al*.^27^ did not find any dramatic change in feeding habits and trophic interactions of consumers, revealing their high trophic plasticity. Although the mixing model did not highlight a shift in consumer diet, it indicated an increased use of SOM for nearly half the herbivores and detritivores (i.e. 4 out of 10 species). This trophic plasticity can be due to a higher SOM quality (i.e. it was much more ^13^C-depleted and had a lower C/N ratio, suggesting a higher contribution of more labile detritus to bulk sedimentary organic matter) and availability to consumers at CO_2_-enriched sites (i.e. a higher deposition of fine sediment on macrophytes was visually observed, authors’ personal observation). However, the mixing model also indicated a high level of uncertainty, which is intrinsic to the model itself^48^ and Bayesian statistics^48, 49^, with very wide credibility intervals indicating a wide spectrum of possible solutions.

Reduction in upper trophic levels was suggested by the truncation of carnivores. Our observations of reduced trophic diversity and redundancy, as derived from isotopic metrics, support the general hypothesis of OA-driven food web simplification. This simplification is usually reflected in an overall simplification of the benthic community, as also observed in other vent systems^11, 19, 23, 50^. Marine communities are well known to simplify their structure when dominated by human activities^51^, particularly where water quality is reduced^52^, including resource enhancement by eutrophication^53, 54^. The reason for observed outbreaks of herbivores^23^, may be explained by this truncation of their predators. Whilst CO_2_ may indirectly boost herbivore populations, this population growth may not be suppressed by intensifying predation.

Our results suggest a dual control on food web structure in CO_2_ vents as a function of bottom-up change (i.e. resource-effect of CO_2_ enrichment) and top-down change (i.e. stressor-effect of OA). Tolerant species were selected under CO_2_ enrichment, leading to higher abundance of fleshy primary producers through a bottom-up control (i.e. CO_2_ as a resource-effect), and to loss of carnivores, which are generally less tolerant to OA^19^. The resulting lack of top-down control (i.e. stressor-effect) failed to suppress bottom-up forces so that herbivore and detritivore abundance boomed. The greater availability of primary producers of higher nutritional quality neither amplified their trophic role nor was hampered by herbivore pressure. Herbivores also failed to suppress bottom-up forces.

The shifting balance between the propagation of resources and their consumption led to simplification of the community and food web structure. The reduced assemblage of motile invertebrates was characterized by a shortened food web that shifted towards greater abundances of herbivores and detritivores and drastic reduction in carnivores that lowered overall trophic diversity. Such assessments are challenging in ocean acidification research because of the difficulty of replicating complex ecological systems in mesocosms and replicating CO_2_ sites where ecological complexity exists naturally. As such, our relatively simplified field observations provide assistance to conceptual development of models about change and stasis in a high CO_2_ world. These results support the conceptual model in which CO_2_ enrichment acts as a resource (i.e. boosted primary productivity and herbivore responses) and a stressor driving lower biodiversity of motile invertebrates with dominance of tolerant and opportunistic species within a simplified food web structure.

Overall, this study not only demonstrates the transfer of CO_2_ enrichment from plants to herbivores through consumption (i.e. CO_2_ as a resource-effect or bottom-up control), but also questions whether predators can compensate for ensuing herbivore outbreaks to stabilize communities (OA as a stressor-effect or top-down control)^8, 44, 54^. Given the key role of biodiversity and trophic function in preserving ecosystem stability^30, 51^, their alterations could have important implications on the ecosystem functioning^55^ and maintenance of ecosystem services^31, 32, 56^.

## Methods

### Study area

Vulcano Island (Italy) is one of seven islands belonging to the Aeolian Archipelago, situated in the Southern Tyrrhenian Sea (Mediterranean Sea, North-Eastern Sicily). The whole archipelago has a volcanic origin and was generated about 1 Ma ago by subduction processes in the Southern Tyrrhenian sea floor, involving the sliding of the Ionian lithosphere beneath the Calabrian Arc^57, 58^. Among the several submerged CO_2_ vents occurring around the archipelago, Levante Bay on the eastern coast of Vulcano presents one of the most widely studied and active area. Gas emissions from a primary vent (38°25.057′N; 14°57.599′E) exhibit a pH gradient of 6.40 to 8.16^59^ that runs mostly parallel to the northern coast of the bay due to the action of north-westerly winds. According to previous studies^60–62^ the vents are mainly composed of CO_2_ (97–99% vol.), while small H_2_S concentrations (<2.2%) seem to be restricted to the primary vent decreasing sharply with distance^59, 60, 62^ and trace element contamination affects mainly the area at about 150–350 m from the primary vent^63^.

### Sample collection

We selected two sampling sites within Levante Bay following the design of previous studies^47^: a weakly acidified site (pH 7.96), called the CO_2_-enriched site and a reference site with normal pH conditions (pH 8.16), the control site (see Supplementary Fig. 1S). The sites were located about 200 and 500 m respectively from the primary vent, at ca. 1–2 m depth, and were both characterised by mixed seagrass and macroalgal beds. No site-replication was possible, since vent pH gradient and control environmental conditions (depth-related factors and exposure to hydrodynamics) change rapidly when moving farther from the selected sites.

We sampled these sites once during May 2013, collecting motile invertebrates in four replicates within each site (CO_2_-enriched and control) using an airlift sampler on a 30 × 30 cm quadrat^64^. Using suction under constant flow for 1 minute we gathered the samples in a collecting bag (mesh size 400 µm) and subsequently collected by hand all the macrophytes present in the same quadrats. We also sampled additional macroinvertebrate individuals to reach the mass requirement needed for each species to perform stable isotope analysis, by collecting macroalgae and then gently shaking them in large trays. However, due to the low biomass of some of the species collected, we did not perform isotopic analysis on all of the species identified. Before storing invertebrate samples, we kept alive all animals collected overnight to clear their guts.

At each site, we collected seawater samples (10 L) and sediment cores (3 cm Ø) in triplicate to determine the isotopic composition of the particulate (POM) and sedimentary organic matter (SOM), respectively.

### Motile invertebrate community structure

We identified motile invertebrates to the lowest taxonomic level possible by stereomicroscope and counted them to determine density relative to sampling surface. We arranged species and higher taxa (genera, families, etc.) of benthic invertebrates into trophic groups defined according to the current literature: Scipione (2013)^65^ for amphipods, Jumars *et al*.^66^ for polychaetes and Gambi *et al*.^67, 68^ for molluscs. In addition, we calculated the Sorensen similarity index and two biodiversity indexes, Shannon’s diversity (*H’*) and Pielou’s Evenness (*J*) index, according respectively to the following equations (1–3):

1 $${\rm{Sorensen}}\mbox{'}{\rm{s}}\,{\rm{index}}\,\,\,\,{{\rm{S}}}_{{\rm{S}}}=2{\rm{a}}/(2{\rm{a}}+{\rm{b}}+{\rm{c}})$$

where a is the number of species common to both communities, b is the number of species unique to the first community, and c is the number of species unique to the second community;

2 $${\rm{Shannon}}\mbox{'}{\rm{s}}\,{\rm{index}}\,\,H\mbox{'}=-\sum _{i=1}^{R}{p}_{i}{\mathrm{log}}_{2}{p}_{i}$$

where *p*_*i*_ is the number of individuals belonging to the *i*th species, divided by the total number of individuals found in the community sample;

3 $${\rm{Pielou}}\mbox{'}{\rm{s}}\,{\rm{index}}\,\,\,\,J\mbox{'}=H\mbox{'}/H{\mbox{'}}_{{\rm{\max }}}$$

where *H’* is the Shannon’s diversity index and *H’*_max_ the maximum value of *H’*.

### Food quality

We assessed food quality in freeze-dried samples of sediment (with the exceptions of phenolics and fibre content) and macrophytes. Carbon and nitrogen contents were quantified using an elemental analyser (Thermo Flash EA1112) and expressed as %. C/N ratio was used as indicator of the nutritional quality to consumers as reported elsewhere (see ref. 68). We determined total phenolics following the Folin–Ciocalteu method modified by Harrison & Durance (1989)^70^ and Bolser *et al*.^69^, using caffeic acid as standard and expressing results as caffeic acid equivalents (CAE) in mg g DW^−1^. We quantified non-structural carbohydrates using the phenol-sulfuric acid colorimetric method^71^ with glucose as standard, after extraction of soluble sugars in hot ethanol and enzymatic conversion of starch to glucose equivalents. We measured the amount of insoluble fibres as the difference in mass (dry weight) after heating the sample to boiling (100 °C) in neutral detergent for 1 h and successively washing with distilled water, ethanol and acetone following a modified method from Van Soest *et al*.^72^. Results were expressed as % (g of fibres per 100 g of dry biomass).

We used Student t-tests to separately test for differences in food quality variables between sites.

### Stable isotope analysis and metrics of trophic structure

We dried all samples of invertebrates, macrophytes and epiphytes scraped from macrophytes at 60 °C for 48 h to remove the aqueous component. In addition, we measured biomass of macrophytes and their epiphytes (dry weight) by weighing after drying at 60 °C for 48 h. We filtered water samples on precombusted (450 °C, 4 h) filters (GF/F Whatman, pore size 0.45 μm) and then oven dried them at 60 °C for 48 h. We dried the first two centimetres of sediment cores under the same conditions.

After drying, we ground samples to fine powder using a micro mill or a mortar and pestle. We analysed samples using an isotope ratio mass spectrometer (Thermo Delta Plus XP) coupled to an elemental analyser (Thermo Flash EA1112). Prior to δ^13^C analysis, we acidified the samples (HCl 1 M) to allow dissolution of the carbonates present in marine organisms that can potentially alter the carbon isotopic signature. Carbon and nitrogen stable isotope ratios were expressed in δ notation, as parts per thousand deviation from standard reference materials, following the equation (4):

4 $${\rm{\delta }}X=[({{\rm{R}}}_{{\rm{sample}}}-{{\rm{R}}}_{{\rm{standard}}})/{(R}_{{\rm{standard}}})]1000$$

where *X* is ^*13*^ *C* or ^*15*^ *N* and *R* is the relative ^*13*^ *C*/^*12*^ *C* or ^*15*^ *N*/^*14*^ *N* ratio. Reference standards were Vienna Pee Dee Belemnite (vPDB) and atmospheric N_2_ for carbon and nitrogen respectively, with a 0.2‰ analytical precision of replicates for both isotope ratios.

To estimate trophic levels of motile invertebrate species (TL_c_) we used the following equation (5) proposed by Post (2002)^73^:

5 $${{\rm{TL}}}_{{\rm{c}}}=[({{\rm{\delta }}}^{15}{{\rm{N}}}_{{\rm{c}}}-{{\rm{\delta }}}^{15}{{\rm{N}}}_{{\rm{b}}})/{{\rm{\Delta }}}_{{\rm{n}}}]+{\rm{\lambda }}$$

where δ^15^N_c_ and δ^15^N_b_ are the nitrogen isotopic signatures of the consumer and the baseline reference species respectively; Δ_n_ is the expected enrichment (isotopic fractionation) in δ^15^N per trophic level, and λ is the trophic level of the species used as baseline. The calculation of trophic level has intrinsic limitations and requires assumptions in the choice of a proper baseline and nitrogen isotopic fractionation value, which may be variable among species and individuals^74^. We chose primary consumers showing the lowest δ^15^N values (i.e., amphipods of the Dexaminidae family) as baseline, according to recommendation provided by Mancinelli *et al*.^75^, and their trophic level (λ) was therefore set as 2. As most species analysed belong to low ranks of the trophic hierarchy, we assumed an isotopic fractionation value of 2.5‰, as determined for herbivores by Vander Zanden & Rasmussen (2001)^76^.

In order to characterise the trophic structure at the CO_2_-enriched and control sites, we estimated community-wide metrics from consumer isotopic data according to Jackson *et al*.^77^, using the R package SIAR (Stable Isotope Analysis in R)^78^. We used the following metrics to describe the trophic structure in terms of trophic diversity according to Layman *et al*.^79^: i) δ^15^N Range (NR) is the difference between the most enriched and most depleted δ^15^N values and provides information on the trophic length; ii) δ^13^C Range (CR) is the difference between the most enriched and the most depleted δ^13^C values and estimates the diversity of basal resources exploited; iii) mean Distance to Centroid (CD) is the average Euclidean distance of each species to the centroid δ^13^C-δ^15^N and represents the trophic diversity and species spacing within the isotopic space; iv) mean Nearest Neighbour Distance (NND) is expressed as the Euclidean distance of each species to the nearest neighbour and measures species density and packing within the community, given by the proximity of each species to another within the same isotopic space (trophic redundancy); and v) Standard Deviation of the Nearest Neighbour Distance (SDNND) provides information on the evenness of species packing.

In addition, according to Jackson *et al*.^77^, we estimated corrected Standard Ellipse Area (SEAc) by Bayesian inference using SIBER package (Stable Isotope Bayesian Ellipses in R) of SIAR in R, which allows to obtain an accurate measure of the isotopic niche width that is not biased by the difference in sampling size between sites.

We estimated the contribution of food sources to each consumer species through Bayesian mixing models, using the aforementioned SIAR package. We considered four main sources of organic matter contributing to the primary consumer diet: seagrasses, algae, epiphytes and SOM. We *a priori* combined all the ecologically related species in each corresponding group (seagrasses and algae) after testing for non-statistically significant differences (non-parametric Kruskal-Wallis test and multiple comparison test after Kruskal-Wallis). This allowed us to reduce the number of sources and hence perform a more robust analysis than using individual species, which generally leads to more constrained and wide solutions^80^. Trophic enrichment factors (TEFs) specific to herbivores were 2.5‰ ±2.5 for δ^15^N and 0.47‰ ±1.23 for δ^13^C following Vander Zanden & Rasmussen (2001)^76^.

The model we ran in SIAR output a dataset of the highest density regions (HDRs) for all parameters examined, including: mean (the mean of all possible solutions), mode (the solution with the highest probability) and 95% credible interval (CI, corresponding to the low 95th percentile – minimum contribution – and high 95th percentile – maximum contribution).

### Data Availability

All data generated or analysed during this study are included in this published article (and its Supplementary Information files).

## Electronic supplementary material

Supplementary tables and figures
