# Supplementary material for: Ocean acidification as a driver of community simplification via the collapse of higher-order and rise of lower-order consumers
Source: Sci Rep. 2017 Jun 22;7:4018. doi: 10.1038/s41598-017-03802-w (PMC5481442; doi:10.1038/s41598-017-03802-w)
Supplement: Supplementary file 1 — Supplementary tables and figures [file 41598_2017_3802_MOESM1_ESM.pdf]

Ocean acidification as a driver of community simplification via the collapse of higher-order and rise of lower-order consumers

Vizzini S.[1,2], Martínez-Crego B.[3], Andolina C.[1,4], Massa-Gallucci A.[5], Connell S.D.[6], Gambi M.C.[5]

Table 1S. List of the taxa found at the a) control and b) CO_2-enriched sites.

a) control site

Taxon	Species
**Crustacea Amphipoda**	*Caprella acanthifera*
	Dexaminidae
	Hyale sp.
	Phtisica marina
	Pseudoprotella phasma
	Sunampithoe pelagica
**Crustacea Cumacea**	spp.
**Crustacea Decapoda**	*Cestopagurus timidus*
	Hippolytidae
	Pagurus anachoretus
**Crustacea Isopoda**	*Dynamene edwardsi*
	Idotea sp.
**Crustacea Tanaidacea**	*Apseudes latreilli*
	Apseudidae
	Chondrochelia savignyi
**Echinodermata**	*Amphipholis squamata*
**Hexapoda**	Chironomidae larvae
**Mollusca Bivalvia**	*Lucinella divaricata*
**Mollusca Gastropoda**	*Bittium reticulatum*
	Cerithium vulgatum
	Columbella rustica
	Cylichna cylindracea
	Eulima sp.
	Gibbula ardens
	Nassarius sp.
	Philine sp.
	Smaragdia viridis
**Nemertea**	spp.
**Polychaeta**	Exogoninae spp.
	Polyophthalmus pictus

	Spionidae
	Syllis prolifera
**Total taxa**	**32**

b) **CO_2-enriched site**

**Taxon**	**Species**
**Crustacea Amphipoda**	*Ampithoe ramondi*
	Caprella acanthifera
	Dexaminidae
	Erichthonius sp.
	Hyale sp.
	Sunampithoe pelagica
**Crustacea Decapoda**	*Upogebia* sp.
**Crustacea Isopoda**	*Dynamene edwardsi*
**Crustacea Tanaidacea**	*Apseudes latreilli*
	Chondrochelia savignyi
**Echinodermata**	*Amphiura filifomis*
	Holothuria sp. juvenile
**Hexapoda**	Chironomidae larvae
**Mollusca Gastropoda**	*Alvania* spp.
	Rissoidae
**Nemertea**	spp.
**Polychaeta**	*Amphiglena mediterranea*
	Paraonidae
	Platynereis cfr *dumerilii*
	Polyophthalmus pictus
	Spionidae
	Syllis prolifera
	Syllidae spp.
**Total taxa**	**23**

Table 2S. Estimates of Bayesian mixing models relative to the control (a) and CO_2-enriched (b) sites. Species are grouped according to their feeding group: filter-feeders (FF), herbivores (H) and herbivores/detritivores (H/D). Contributions are expressed as low and high 95th percentile proportion that represent the lower and the upper limit of the range of possible solutions given by the mixing model output of the density region, with relative mode and mean.

a)

**control site**		**Seagrasses**			**Algae**			**Epiphytes**			**SOM**		
**Feeding group / Species**		Low - High 95%	Mode	Mean	Low - High 95%	Mode	Mean	Low - High 95%	Mode	Mean	Low - High 95%	Mode	Mean
**FF**	***Amphiglena mediterranea***	0 - 0.48	0.28	0.25	0 - 0.48	0.27	0.25	0 - 0.49	0.27	0.25	0 - 0.49	0.27	0.25
**H**	***Columbella rustica***	0 - 0.48	0.29	0.25	0 - 0.50	0.27	0.25	0 - 0.49	0.28	0.25	0 - 0.48	0.28	0.25
	**Hippolytidae**	0 - 0.36	0.03	0.15	0.03 - 0.65	0.32	0.35	0.03 - 0.50	0.31	0.29	0 - 0.41	0.25	0.21
	Platynereis* cfr *dumerilii	0 - 0.44	0.25	0.22	0 - 0.53	0.30	0.28	0 - 0.49	0.29	0.26	0 - 0.46	0.28	0.24
**H/D**	***Caprella acanthifera***	0 - 0.46	0.26	0.24	0 - 0.51	0.29	0.26	0 - 0.47	0.29	0.24	0 - 0.50	0.31	0.27
	**Dexaminidae**	0 - 0.44	0.25	0.21	0 - 0.55	0.32	0.29	0 - 0.44	0.22	0.21	0.01 - 0.52	0.31	0.29
	Dynamene edwardsi	0 - 0.46	0.26	0.23	0 - 0.52	0.30	0.27	0 - 0.46	0.27	0.23	0 - 0.51	0.30	0.27
	***Hyale* sp.**	0 - 0.43	0.04	0.20	0 - 0.57	0.31	0.30	0 - 0.45	0.16	0.21	0.01 - 0.53	0.31	0.29
	Chondrochelia savignyi	0 - 0.38	0.03	0.16	0.02 - 0.67	0.32	0.35	0 - 0.40	0.13	0.20	0.01 - 0.51	0.31	0.29
	Sunamphitoe pelagica	0 - 0.48	0.27	0.24	0 - 0.49	0.27	0.25	0 - 0.48	0.28	0.25	0 - 0.49	0.28	0.25

b)

**CO_2-enriched site**		**Seagrasses**			**Algae**			**Epiphytes**			**SOM**		
**Feeding group / Species**		Low - High 95%	Mode	Mean	Low - High 95%	Mode	Mean	Low - High 95%	Mode	Mean	Low - High 95%	Mode	Mean
**FF**	***Amphiglena mediterranea***	0 - 0.48	0.27	0.24	0 - 0.49	0.29	0.25	0 - 0.49	0.28	0.25	0 - 0.50	0.29	0.26

H	***Columbella rustica***	0 - 0.36	0.03	0.15	0 - 0.46	0.28	0.24	0 - 0.44	0.27	0.22	0.06 - 0.81	0.33	0.39
	**Hippolytidae**	0 - 0.43	0.04	0.20	0 - 0.49	0.29	0.26	0 - 0.47	0.27	0.24	0 - 0.59	0.31	0.30
	Platynereis* cfr *dumerilii	0 - 0.26	0.02	0.10	0 - 0.48	0.28	0.25	0 - 0.43	0.21	0.21	0.19 - 0.70	0.43	0.44
H/D	***Caprella acanthifera***	0 - 0.42	0.04	0.20	0 - 0.48	0.28	0.25	0 - 0.47	0.31	0.23	0 - 0.62	0.31	0.32
	**Dexaminidae**	0 - 0.46	0.29	0.23	0 - 0.49	0.27	0.25	0 - 0.47	0.28	0.24	0 - 0.52	0.29	0.28
	Dynamene edwardsi	0 - 0.46	0.27	0.23	0 - 0.48	0.30	0.25	0 - 0.47	0.27	0.25	0 - 0.52	0.29	0.27
	***Hyale*sp.**	0 - 0.42	0.04	0.19	0 - 0.48	0.26	0.25	0 - 0.47	0.27	0.23	0 - 0.66	0.32	0.34
	Chondrochelia savignyi	0 - 0.37	0.03	0.15	0 - 0.47	0.26	0.24	0 - 0.44	0.14	0.21	0.05 - 0.80	0.34	0.40
	Sunamphitoe pelagica	0 - 0.45	0.26	0.22	0 - 0.49	0.29	0.25	0 - 0.47	0.26	0.24	0 - 0.54	0.30	0.28

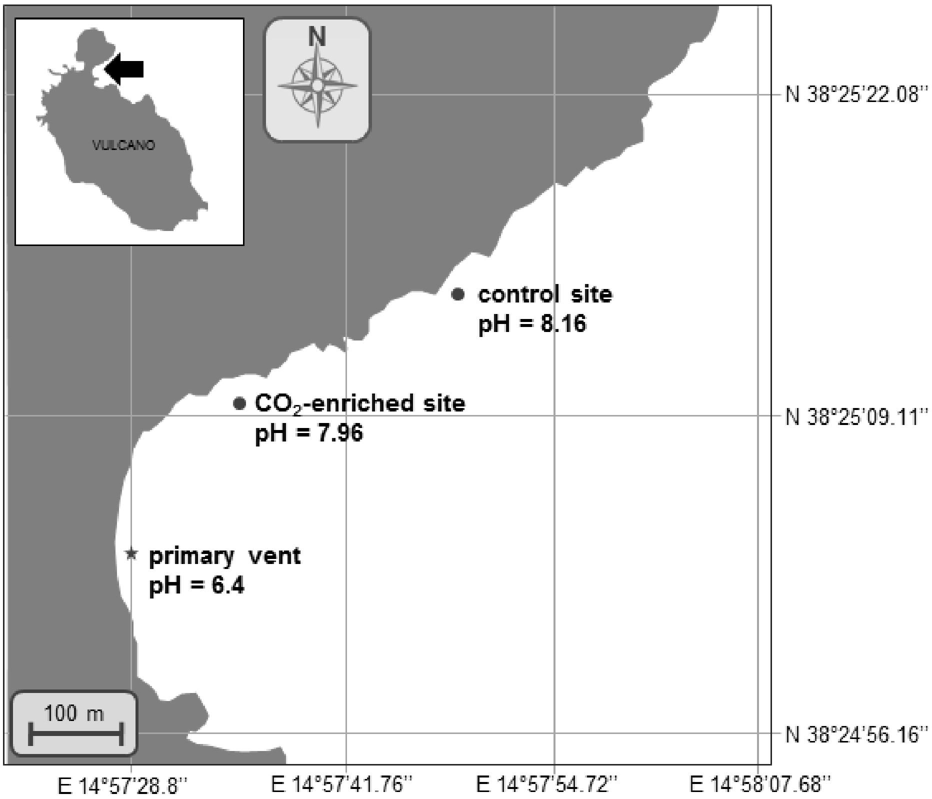

Figure 1S. Map of the study area. Location and mean pH values of the study sites (control and CO_2-enriched) in relation to the primary vent at the Levante Bay of Vulcano Island (Windows Power Point 2013 drawing based on maps from ArcGIS vers. 9.1).

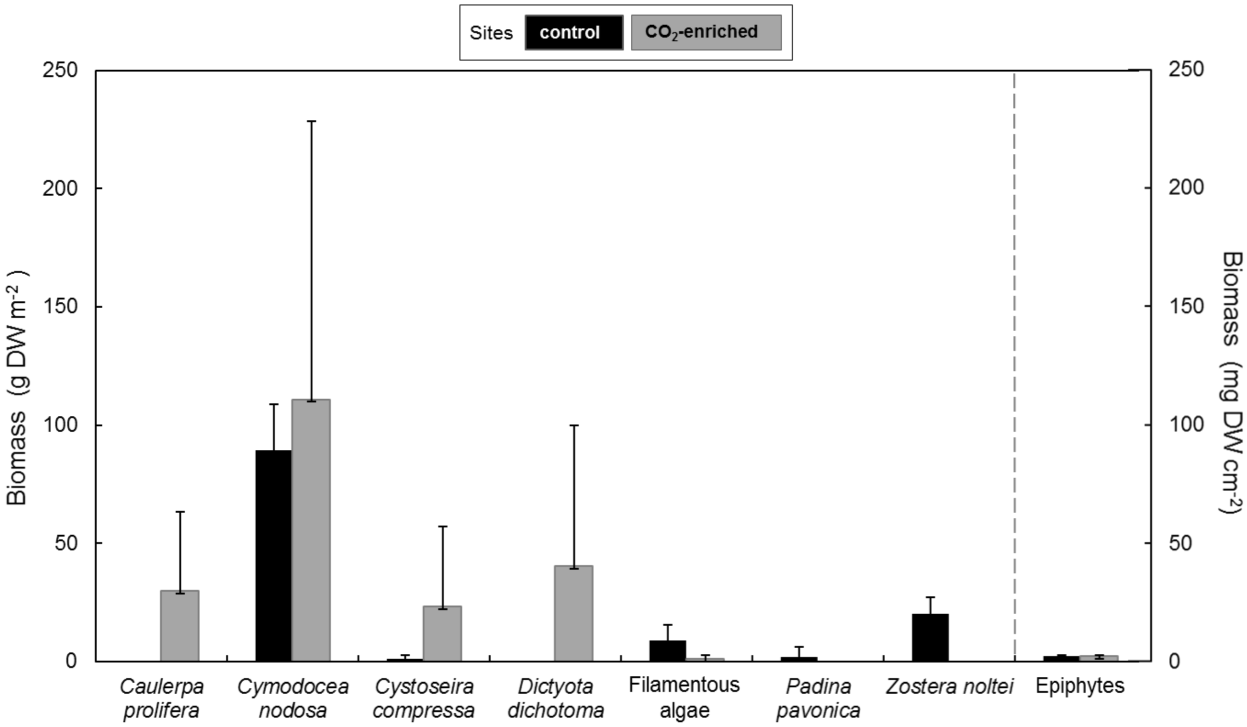

Figure 2S. Abundance of macrophytes and epiphytes at the control (black) and CO_2-enriched (grey) sites. Biomass of macrophyte species (g DW m^{-2}; mean ± standard deviation) and epiphytes (mg DW cm^{-2}; mean ± standard deviation).
